# Performance of lunar shell structure for moonbase subjected to low gravity coupled with changing temperature

**DOI:** 10.1016/j.fmre.2024.08.005

**Published:** 2024-09-03

**Authors:** Yuyue Gao, Yan Zhou, Shanshan Cheng, Wenbin Han, Cheng Zhou, Lieyun Ding

**Affiliations:** a National Center of Technology Innovation for Digital Construction, Huazhong University of Science and Technology, Wuhan 430074, China; b School of Civil and Hydraulic Engineering, Huazhong University of Science and Technology, Wuhan 430074, China

**Keywords:** Lunar shell structure, Thermal analysis, Thermal stress, Heat loss, Temperature field

## Abstract

Temperature variations on the lunar surface can cause significant thermal stress and increase energy consumption within a lunar base. Thus, studying the thermal behavior of lunar structures over time and space is crucial. This study utilizes classical thermomechanical coupling simulations to develop a comprehensive methodology for assessing the thermal performance of lunar shell structures. The model incorporates realistic assumptions about critical loads and boundary conditions and uses the real properties of regolith-based construction materials as input parameters. Using this method, the study simulates 36 scenarios for shell structures, including three types of structures, four latitudes, and three time periods. The temperature field, heat loss, and thermal stress for each scenario are calculated, and the overall trends and specific cases are analyzed. These results can inform further recommendations for the architectural design of lunar shell structures and the selection of construction materials.

## Graphical abstract

**Figure:**
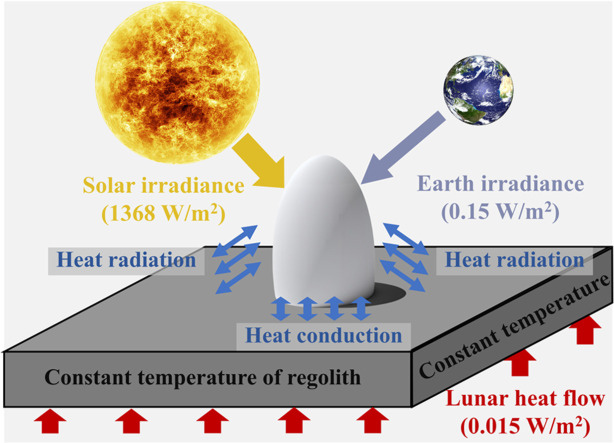

## 1 Introduction

Human space exploration represents a continuous process of growth and advancement. Starting in the 1950s, the United States and the Soviet Union engaged in an exploration competition that led to the first wave of lunar exploration through the Apollo and Luna programs. As we entered the 21st century, more countries proposed the idea of establishing long-term residency in extraterrestrial space and developed plans for lunar bases. NASA's Artemis program, for instance, aims to establish a lunar base camp as a stepping stone for Mars exploration [1]. China has also launched its own manned lunar exploration project with the goal of achieving a Chinese landing on the moon by 2030 [2]. Lunar construction has now become a crucial area of research for further exploration of deep space [3,4]. Digital design and intelligent construction methods, which are prevalent in the field of construction on Earth [[5], [6], [7], [8]], are gradually showing their potential for extraterrestrial structures.

The main objective of moonbase construction is to provide a stable internal environment for crew members and important equipment. Therefore, the design of shapes and configurations plays a vital role. Some typical structures for moonbase include the lunar outpost and the moon village by ESA [9], Xuanwu Station by China [10], and the Lunar Lantern by ICON and NASA [11].

The service of lunar habitat structures faces significant challenges, particularly in relation to the extreme temperatures on the lunar surface. Unlike on Earth, there is no atmosphere to act as an energy-absorbing buffer, resulting in the maximum solar irradiation on the lunar surface being almost equivalent to the solar constant [12]. This means that the highest temperature on the lunar surface can reach 397 K, while the lowest temperature can decrease to as low as 100 K [13,14]. These extreme temperature cycles have a major impact on lunar surface structures in two main ways. First, the thermal stress inside the structure can be significant and can vary as the temperature increases. This thermal stress can cause the growth of microcracks within the structure, potentially leading to structural damage [15,16]. Construction materials are also susceptible to thermal fatigue due to variable thermal stress [17]. Second, maintaining a stable internal temperature in the lunar environment requires a high level of energy consumption, which is both uneconomical and unsustainable. Therefore, one of the key design goals for lunar structures is to achieve good thermal insulation to minimize energy consumption [18].

Previous studies have conducted preliminary explorations into the temperature field distribution and internal thermal stress of lunar structures. For example, Steiner et al. [19] performed a transient heat transfer analysis on a specifically designed layered structure made of composite materials to evaluate its insulation capacity. Moreover, Mottaghi et al. [20] conducted a steady-state heat transfer analysis on an igloo-shaped structure located at the South Pole of the Moon. This structure was made of magnesium alloy and covered with sandbags filled with regolith for shielding. Tripathi et al. [21], on the other hand, used an explicit finite difference scheme to infer the temperature distribution of a dome habitat made of sintered regolith. In their analysis, they considered necessary heat sources and heat sinks but relied on property data from the literature rather than unified preparation parameters. However, relatively limited research has been conducted on the thermal stress analysis of lunar structures. Steinberg et al. [22] derived a simplified formula for calculating thermal stress, assuming the complete constraint of material thermal expansion. Brown et al. [23] performed a sequentially coupled thermal stress analysis on a structure made of aluminum and covered with regolith.

It is important to note that there are limitations to the thermal performance evaluation of lunar structures. First, most of the researched structures are made of alloys rather than utilizing in situ resources on the Moon. This can result in heavy loads for Earth-moon transportation and subsequently increase construction costs. The thermophysical properties of regolith-based materials differ greatly from those of metals, rendering the results of studies on alloy-based structures of little reference value. As a result, further modeling and analysis of in situ construction materials are necessary. Second, there is a lack of comparisons regarding the thermal performance of different architectural designs. The previously discussed structures are considered under specific working conditions; therefore, the spatial and temporal variations in the temperature field and thermal stress remain unclear. Third, there is a need for comprehensive and reasonable boundary condition settings in simulation models. Some studies fail to consider the direction of radiation incidence, while others overlook the influence of structural shadow areas.

To address these limitations, this research proposes a comprehensive evaluation methodology for the thermal performance of lunar shell structures. As outlined in Section 2, specific shell structures for moonbase are proposed as the focus of analysis, with regolith-based construction materials characterized as model inputs. Realistic and comprehensive assumptions regarding key loads and boundary conditions are applied in the model. In Section 3, a total of 36 scenarios of shell structures are simulated to investigate their spatial and temporal thermal behavior. The temperature field, heat loss, and thermal stress for each scenario are calculated and further analyzed.

## 2 Methodology

### 2.1 Architectural design for lunar shell structures

For the examination of the thermal performance of moonbase, a specific type of shell structure is proposed as the research subject. The shell structure consists of an egg-shaped main structure and a catenary roof. The egg-shaped main structure is designed to ensure a uniform stress distribution and provide a larger habitable space [24]. The catenary curve of the roof refers to the shape of a soft and uniform chain under gravity, which can deliver excellent stability and superior stress distribution [25]. In practical applications, the structure will be equipped with inflatable airbags on the inside to ensure airtightness and provide a stable environment for the crew.

**Fig. 1:**
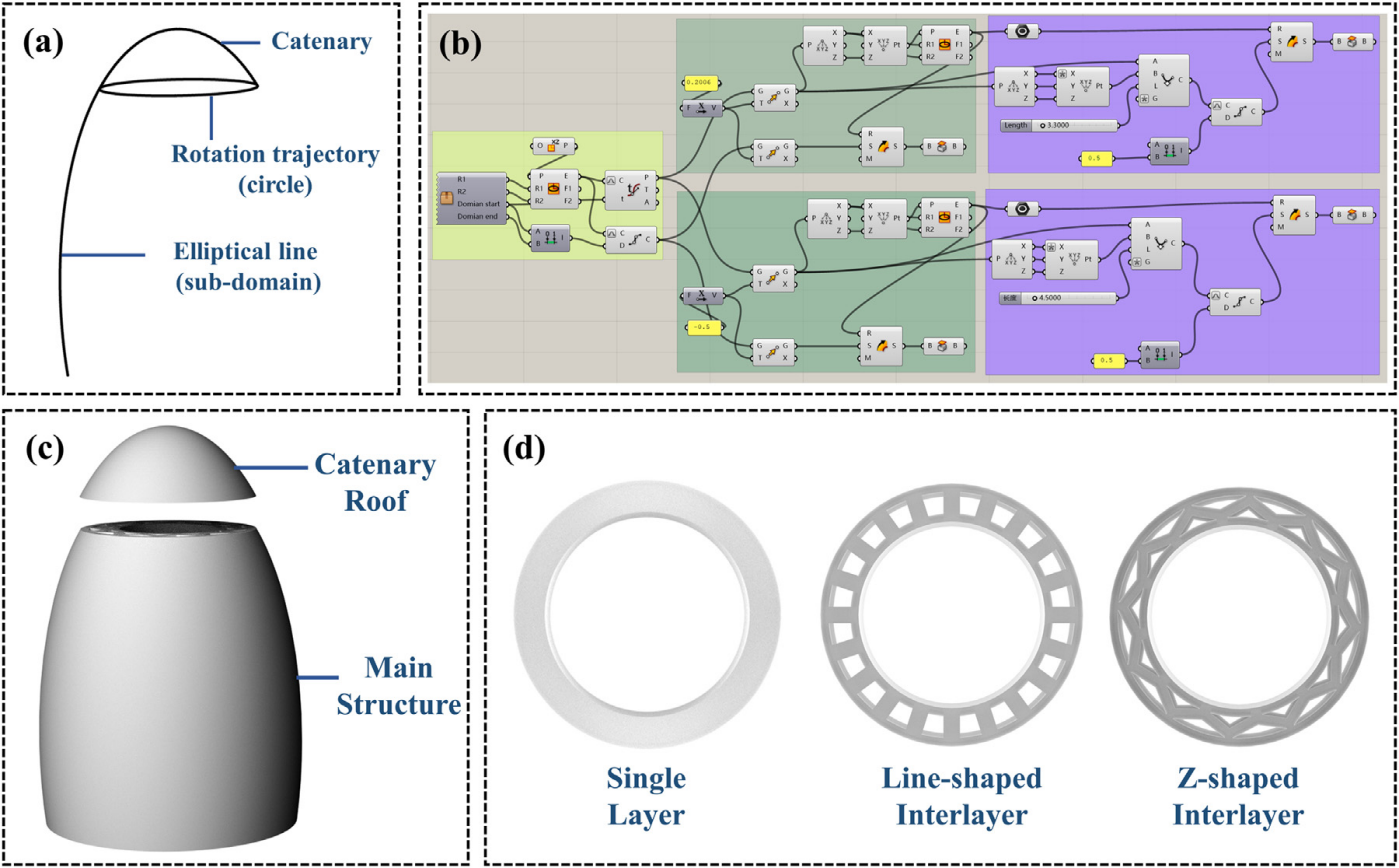
**Architectural design of the proposed lunar shell structure**.

The specific design process for the proposed structure is depicted in Fig. 1. The main structure is formed by a subdomain of an elliptical line, which rotates around a circular trajectory to create the inner surface of the structure. Similarly, the roof structure is formed by dividing the catenary roof in half and rotating it around the same circular trajectory to create the inner surface of the roof, as shown in Fig. 1(a). The parameters of the catenary roof are adjusted to ensure a smooth interface between the roof and the main structure. The inner surface of the structure can be translated outward to form the outer surface, with the distance between them representing the thickness of the structure. This design process is implemented using the parametric design tools Grasshopper (GH) and Rhino, as shown in Fig. 1(b). The resulting structure is illustrated in Fig. 1(c).

Building upon this foundation, the design of the walls of the shell structure is further modified to achieve lightweight design and improve the thermal insulation performance of the structure. A cross-sectional view of the modified structure is shown in Fig. 1(d). The basic architecture consists of a single-layer structure with no interlayer. In contrast, the other two architectures include a line-shaped interlayer and a Z-shaped interlayer, which are expected to enhance the thermal insulation performance by incorporating hollow spaces. The specific parameters of these three structures are described below.

#### 2.1.1 Single-shell structure

The single-shell structure serves as the foundation, featuring no interlayer or complex design. It is used as the control group for comparing and analyzing the performance of the other two structures. Therefore, the design parameters must be carefully determined. To provide a comfortable working and living environment, the confined space should be larger than $40\;m^{3}$ [26]. The major and minor axes of the inner surface's elliptical line are determined as 12.5m and 5m, respectively. Accordingly, the height of the single-shell structure is 8.52m, and the diameter at the widest point is 5.5m. In addition to providing ample interior space, the structure also needs to protect against space radiation and temperature fluctuations. Therefore, the shell thickness is determined as 0.7m, aiming to shield against neutron and proton radiation from Galactic Cosmic Rays [27,28].

#### 2.1.2 Double-shell structure with a line-shaped interlayer

Distinct from a single-layer configuration, the structure is enhanced to feature a double-shell design, characterized by a line-shaped interlayer. With internal airbags in mind, the catenary roof is solely affixed to the outer layer. The line-shaped interlayer is intended to offer robust support for the outer layer, thereby ensuring structural stability. Additionally, the presence of voids significantly reduces the heat conduction area between the two layers, leading to improved thermal insulation.

In determining the specific design parameters of the double-shell structure, the concept of variable control is predominantly employed to facilitate more meaningful comparisons and discussions. This approach is manifested in three key aspects. First, the inner surface of the primary structure remains consistent between the double-shell and single-layer configurations, providing identical suitable space. Second, there is a focus on maintaining similar material consumption, aligning with the principle of lightweight design. Third, the overall height of the structure is kept comparable to ensure a uniform appearance between the two designs. Within the constraints of these three controlled variables, the architecture of the double-shell structure with a line-shaped interlayer is established. As outlined in Table 1, the height and material consumption closely align with those of the single layer. Additionally, when determining the layer thickness, consideration is given to the protective performance of the structure. Specifically, the thicknesses of the inter- and outer layers are both 0.2 m. The thickness of the interlayer is 0.4 m, and the number of interlayers is 20. Subsequently, the double-shell structure with a line-shaped interlayer is abbreviated as the line-shaped structure.

**Table 1:** **Structural parameters of the lunar shell structure**.

Architectural design	Height (m)	Thickness (m)	Consumption of building materials (m^3^)
Single-shell structure	8.52	0.7	80.23
Line-shaped structure	8.76	Inner layer: 0.2 Outer layer: 0.2 Interlayer: 0.4	77.86
Z-shaped structure	8.76	Inner layer: 0.2 Outer layer: 0.2 Interlayer: 0.3	78.57

#### 2.1.3 Double-shell structure with a Z-shaped interlayer

Another proposed double-shell structure is the Z-shaped structure, which differs from the line-shaped structure in the shape of its interlayer. The Z-shaped interlayer has fewer contact fulcrums, resulting in a smaller thermal conduction area. Similar to the line-shaped structure, the variable controlling concept is applied to determine the parameters of the Z-shaped structure. Both the inner and outer surfaces of the Z-shaped structure are identical to those of the line-shaped structure, providing the same habitable space and maintaining the same appearance. The thickness of layers is adjusted to control the consumption of building materials and provide enough protection at the same time, which is finally determined as 0.2m, 0.2m and 0.3m.

### 2.2 Material properties of the lunar shell structure

In addition to determining the architectural design of lunar shell structures, it is important to clarify the properties of construction materials. These properties are essential inputs for thermal simulation analysis. Previous research on thermal simulation for lunar bases has mostly focused on metal structures. These structures are typically designed to be covered with a certain thickness of lunar regolith to improve thermal insulation performance. However, it is worth noting that metal structures are difficult to construct on site and must be transported from the Earth. This poses a significant burden on Earth–Moon Transportation due to the size and weight of these structures. To promote more sustainable development, it is necessary to consider in situ resource utilization and construction. Therefore, it is important to simulate the thermal performance of in situ constructed structures and utilize regolith-based construction materials in thermal simulation analysis.

When performing coupled thermomechanical analysis, it is crucial to characterize construction materials in terms of their thermophysical and mechanical properties. Regolith-based construction materials have been developed in various previous studies; however, very few materials have been reported to have all the required parameters for simulation [29]. To address this deficiency, this study utilizes the technique of selective laser sintering (SLS) to form construction materials and test important properties of lunar regolith samples. SLS is an additive manufacturing process that uses lasers with high power to sinter powder materials layer by layer, resulting in the formation of three-dimensional objects [30]. The CUG-1A lunar regolith simulant [31], which was sieved to a pore size of 150 µm and mixed with 15% epoxy resin, was used as the raw material for this research. During the sintering process, the resin melts and quickly solidifies to aggregate the lunar regolith particles. After manufacturing, the green body formed can undergo postheat treatment to remove the resin and enhance its strength.

As a form of additive manufacturing, the SLS technique allows for the printing of diverse architectural shapes, meeting the requirements for extraterrestrial structures. It also boasts a high rate of in situ resource utilization and printing accuracy [32]. Fig. S1 in Supplementary material shows a scaled model of the designed Z-shaped structure that was printed using SLS, confirming the feasibility of printing the designed structure. Additionally, samples for testing are produced and characterized.

#### 2.2.1 Thermophysical properties

**Fig. 2:**
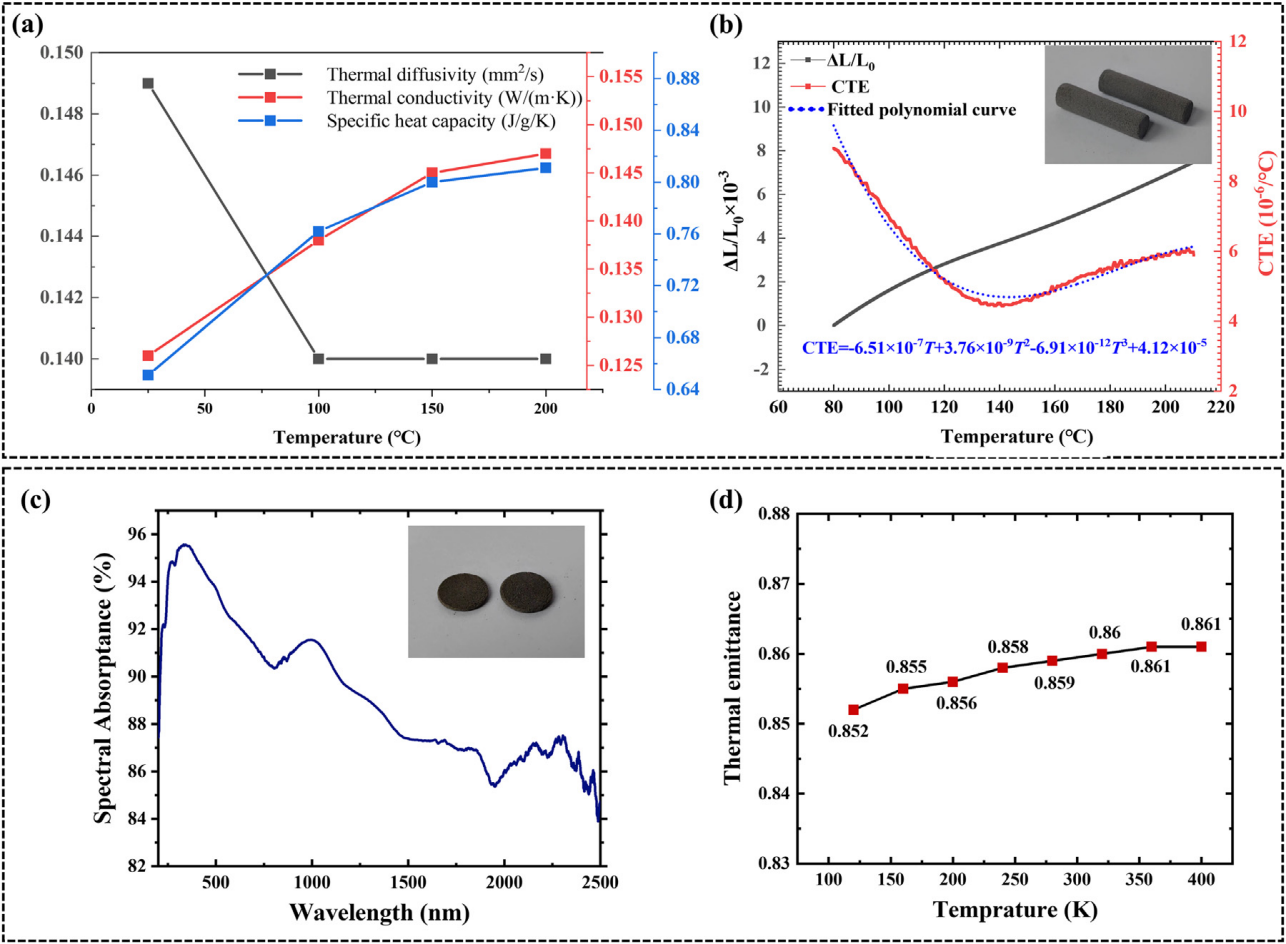
**Thermophysical and optical properties of SLS materials: (a) heat capacity, heat conductivity and thermal diffusivity, (b) CTE, (c) spectral absorptance, and (d) total thermal emittance**.

The thermophysical properties of construction materials have a direct impact on the processes of thermal conduction and thermal radiation, as well as on thermal stress. In terms of thermal conduction, the most important indicators are heat capacity, heat conductivity, and thermal diffusivity. These indicators can be expressed as functions of temperature, as shown in Eq. (1):

(1) K(T)=α(T)c(T)ρ(T)

where T is the temperature, K is the thermal conductivity, α is the thermal diffusivity, c is the heat capacity, and ρ is the density. The above three indicators of the SLS samples are tested within the temperature range of $\left[25{\;}^{\circ }C,200{\;}^{\circ }C\right]$, as shown in Fig. 2(a).

In regard to thermal stress, it is necessary to explore the thermal expansion properties of SLS materials, as thermal stress arises from the restricted expansion of construction materials. The deformation curves of the samples within the temperature range of $\left[80{\;}^{\circ }C,200{\;}^{\circ }C\right]$ are shown in Fig. 2(b). The coefficient of thermal expansion (CTE) is equivalent to the derivative of deformation with respect to temperature. After fitting with a cubic polynomial, the final CTE input into the model is given by Eq. (2):

(2) $$\begin{array}{c} \text{CTE}\left(T\right)=\left\{ \begin{array}{c} 0.00000964608,\;T<80\;\text{°}C\\ -6.51\;\times 10^{-7}T+3.76\;\times 10^{-9}T^{2}-6.91\;\times 10^{-12}T^{3}+4.12\;\times 10^{-5},\\ \;80\;\text{°}C\leq T\leq 200\;\text{°}C \end{array}\right. \end{array}$$

In addition to thermal conduction and thermal expansion, it is essential to consider thermal radiation. Therefore, the spectral reflectance R(λ) [33] of the SLS materials within the wavelength range of [200nm,2500nm] was tested using a spectrophotometer (SolidSpec-3700), and the results are shown in Fig. 2(c). SLS materials can be regarded as opaque materials, and their spectral absorptance α(λ) can be obtained using Eq. (3):

(3) α(λ)=1−R(λ)

According to Kirchhoff's law, the spectral absorptance α(λ) is equal to the spectral emittance ε(λ). The thermal emittance at temperature T can be calculated by

(4) $$\varepsilon \left(T\right)=\frac{\int_{\lambda_{\text{min}}}^{\lambda_{\text{max}}}\varepsilon \left(\lambda \right)\times I_{\text{BB},T}\left(\lambda \right)d\left(\lambda \right)}{\int_{\lambda_{\text{min}}}^{\lambda_{\text{max}}}I_{\text{BB},T}\left(\lambda \right)d\left(\lambda \right)}$$

where $\lambda_{\text{max}}$ is 2500nm, $\lambda_{\text{min}}$ is 200nm, and $I_{\text{BB},T}\left(\lambda \right)$ is the blackbody irradiance at temperature T. The resulting thermal emittance of the SLS material is shown in Fig. 2(d). It is evident that there is minimal numerical change within the temperature range of [125K,400K]. Consequently, the thermal emittance is determined to be the average value of 0.85775 in the simulation model. Considering that regolith particles can migrate to the outer surface of the shell structure, the emittance of the outer surface is set to 0.93 [20], which is equal to that of the lunar regolith.

As for the thermophysical properties of regolith, the heat capacity, as well as the heat conductivity is set to the same value with the research of Zhou et al. [34], as shown in Table 2. In fact, the parameters we used in the paper can only serve as a reference value due to the lack of enough data and the regional differences in lunar regolith performance has been ignored.

**Table 2:** **Thermophysical properties of regolith**.

Temperature (K)	Specific capacity (J/kg K^-1^)	Heat conductivity (W/m K^-1^)
100	275.7	0.0007
150	433.9	0.0008
250	872.4	0.0011
300	758.1	0.0014
350	848.9	0.0017

#### 2.2.2 Mechanical properties

For the mechanical properties, the important indicators include the density, strength, elastic modulus, and Poisson's ratio. The compressive stress‒strain curve of the ϕ10mm×20mm cylindrical sample is tested. Based on this curve, the compressive strength of the sample is calculated to be 5.46 MPa, and the elastic modulus is determined to be 431.84 MPa, as presented in Table 3. The value of Poisson's ratio is taken from the literature and set to 0.2 [35].

**Table 3:** **Mechanical properties of the SLS samples**.

Density	Compressive Strength	Elastic Modulus
1.294 g/cm^3^	5.46 MPa	431.84 MPa

### 2.3 Finite element model for the lunar shell structure

The analysis of the thermal performance of the proposed shell structure is the primary objective of this study. The investigation is carried out by examining the structure's response at different locations and different local times. A total of 36 working conditions for a lunar base are calculated, which include 3 types of structures, 4 types of latitudes, and 3 types of time.

#### 2.3.1 Thermal load and boundary conditions

**Fig. 3:**
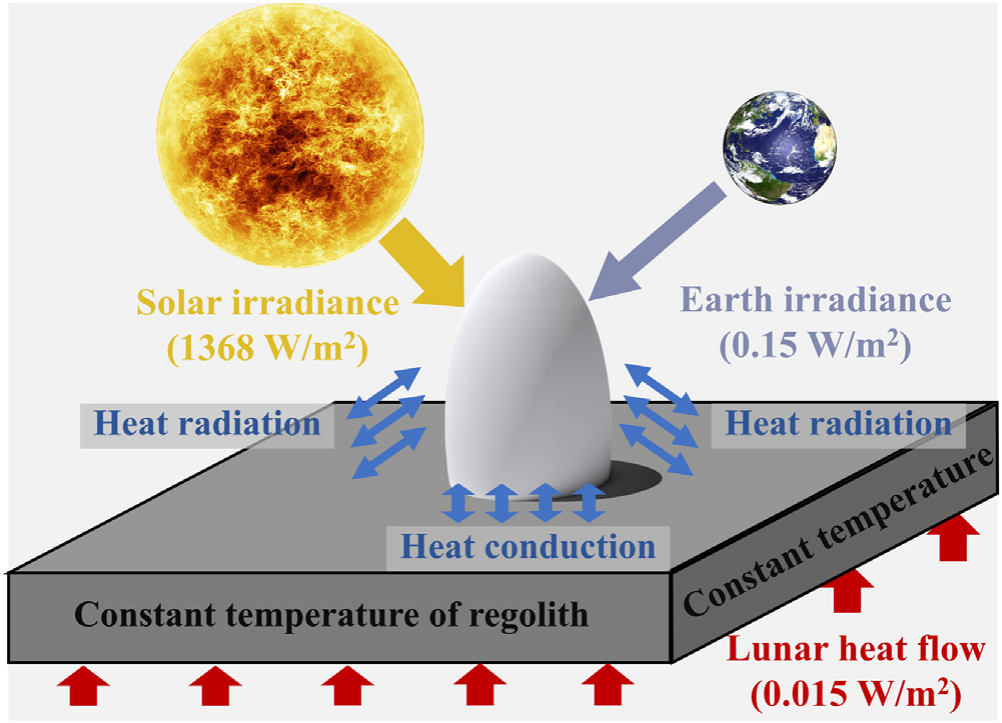
**Thermal load for shell structure on lunar surface**.

The thermal load applied to shell structures can be divided into two main types according to their source. First, the irradiance from the Sun and the Earth, which varies based on location and time. Second, heat exchange between the structure and the lunar regolith, which occurs through radiation and conduction. In addition, the inner surface temperature of the shell structure was maintained at a constant value of 293.5K to ensure a comfortable environment for the crew. In the case of a double shell structure, thermal radiation also occurs between the interlayer surfaces. Notably, the radiation emitted from the innermost surface of the structure, particularly from the inner surface of the catenary roofs of the double-shell structures, is disregarded due to the presence of internal airbags. To simulate the thermal load, a simulation model, depicted in Fig. 3, is created. External radiation is approximated by coming from an infinite distance, mimicking solar and Earth radiation. The lunar regolith is represented by a rectangular cuboid with dimensions of 30m×30m×1m to simulate heat exchange. The large area of the cuboid ensures coverage of most potential heat exchange areas, while the depth is set to 1m since the temperature of the underground regolith is assumed to be constant [36]. To account for heat exchange between the cuboid regolith and other surrounding regolith, the bottom and sides of the cuboid are stipulated to have constant temperatures determined by the empirical formula for lunar surface temperature. For a more precise simulation, a constant heat flow of 0.015W is applied at the bottom of the cuboid, originating from within the moon [37,38].

For solar irradiance, the heat flux is determined as $1368\;W/m^{2}$ according to Huang [39]. Without considering the angle between the white and ecliptic planes, the maximum solar radiation incidence angle at a point on the lunar surface, which refers to the angle between solar rays and the normal of the horizontal plane, is equal to the latitude of that point. Within a lunar day, the incidence angle of solar radiation at a specific point on the moon initially decreases and then increases, ranging from $90^{\circ }$ to the maximum radiation incidence angle, and then increases back to $90^{\circ }$. Assuming that a lunar day consists of 28 days, with 14 days being daytime, and assuming that the solar radiation angle varies uniformly [40], the solar incidence angle at different times and latitudes can be calculated.

Earth irradiance stems from the reflection of solar radiation, which can be determined as $0.15\;W/m^{2}$ according to Song [36]. Taking into account that the Moon revolves around Earth and is locked in by tides, the angle of Earth irradiance can be directly determined by the longitude and latitude of the lunar surface, which remains constant over time. In the proposed model, the angle is assumed to be equal to the latitude of the point where the structure is located.

To determine the temperature of the bottom and sides of the regolith cuboid, a function for the lunar surface temperature needs to be derived. For this purpose, the empirical formula from Mackay [41] and Simonsen [40] is introduced, which can be expressed as

(5) $$T=\left\{ \begin{array}{c} T_{\text{max}}\times \text{sin}{\left(a\times t+b\right)}^{1\;/\;6},\;0\;d\leq t<14\;d\\ 120,\;14\;d\leq t\leq 28\;d \end{array}\right.$$

where $T_{\text{max}}$ refers to the highest temperature at the specific latitude of the Moon, a and b are the coefficients used to adjust the temperature cycle, and t is the time.

The highest temperature $T_{\text{max}}$ is determined by the balance of thermal radiation exchange, indicating that the amount of radiation emitted from the lunar surface is equal to the amount of radiation it receives. This can be described by the following equation:

(6) $$\alpha \text{Scos}\emptyset =\varepsilon \sigma \left({T_{\text{max}}}^{4}-3^{4}\right)$$

where ε refers to the regolith emissivity and α refers to the regolith absorptivity, which are both 0.93.σ represents the Boltzmann constant, and ∅ represents the maximum incidence angle.

According to (5), (6), the temperature formula of the four specific latitudes discussed in this research can be derived as

(7) $$\left\{ \begin{array}{c} T_{\text{latitude}-0^{\circ }}=394.1\sin{\left(12.851d+0.0457\right)}^{1\;/\;6}\\ T_{\text{latitude}-30^{\circ }}=380.2\sin{\left(12.849d+0.0567\right)}^{1\;/\;6}\\ T_{\text{latitude}-60^{\circ }}=331.4\sin{\left(12.839d+0.129\right)}^{1\;/\;6}\\ T_{\text{latitude}-88^{\circ }}=173.0\sin{\left(11.851d+7.022\right)}^{1\;/\;6} \end{array}\right.,d\in \left[0,14\right]$$

The specific value of the above thermal loads is listed in the supplementary material of Table S1.

#### 2.3.2 Mechanical load and boundary conditions

Compared to the aforementioned complex thermal load, the mechanical load is relatively simple. It consists only of the body force of $\frac{1}{6}$ gravity [42]. Since there is an airbag inside the structure, no internal air pressure is considered. The cuboid of the regolith acts as a domain constraint, limiting the displacement and rotation of the shell structure's bottom. This constraint can inhibit the free thermal expansion of construction materials, resulting in thermal stress [43].

#### 2.3.3 Finite element analysis and solution

To assess the thermal performance of the structure, the temperature field and stress field of the model need to be determined. To achieve this, the finite element analysis method is employed. This approach involves the governing equations of thermal radiation, thermal conduction, and coupled thermomechanical analysis. Furthermore, the variation in the lunar surface temperature is sufficiently slow, allowing the thermal behavior of the structure to approximate a steady state.

Regarding thermal radiation, the following surfaces should be considered: the surface of the lunar regolith, the outer surface of the shell structure, and the interlayer inside the double-shell structure. The total irradiance, denoted as $q_{r}$, can be expressed as

(8) $$q_{r}=q_{m}+q_{\text{ext}}+q_{\text{amb}}$$

where $q_{r}$ indicates the whole irradiance, $q_{m}$ is the mutual irradiation from other boundaries in the model, $q_{\text{ext}}$ is the irradiation from external radiation sources, and $q_{\text{amb}}$ is the ambient irradiation [44].

(9) $$q_{\text{ext}}=\sum F_{\text{ext}}q_{0,s}$$

where $F_{\text{ext}}$ refers to the external heat sources view factor, which equals cosθ for a source at infinity, and θ is the incidence angle. $q_{0,s}$ is the heat flux of the directional radiative sources, which corresponds to the solar irradiance and Earth irradiance in the model.

(10) $$q_{\text{amb}}=\varepsilon_{\text{amb}}F_{\text{amb}}e_{b}\left(T_{\text{amb}}\right)$$

where $\varepsilon_{\text{amb}}$ is the ambient emissivity, $F_{\text{amb}}$ is the ambient view factor and $T_{\text{amb}}$is the assumed far-away temperature. In the proposed model, the environments for both the inner layer and outer surface have already been built, so there is no need to apply environmental radiation repeatedly. $T_{\text{amb}}$ is set to the value of 0, and $q_{\text{amb}}$ is also 0.

With the irradiance $q_{r}$ as one of the heat flow inputs, the temperature field of the model can be solved by the following equation:

(11) $$\rho C_{p}\frac{\partial T}{\partial t}+\nabla \left(\tilde{q}+\tilde{q_{r}}\right)=-\alpha T\frac{\text{dS}}{\text{dt}}+Q$$

where ρ is the density, $C_{p}$ is the specific heat capacity, T is the temperature and t is time. For the steady-state analysis, the first item on the left-hand side is 0. In addition, $\tilde{q}$ and $\tilde{q_{r}}$ represent the heat fluxes of thermal conduction and thermal radiation, respectively. α is the coefficient of thermal expansion, and S is the stress tensor. The first item on the right-hand side refers to the thermoelastic damping, which is also 0 in the steady-state analysis. Finally, Q contains an additional heat source. By solving Eq. (11), the temperature field of the proposed model can be obtained.

According to the analysis above, the temperature field is not influenced by the stress field in steady-state analysis. Hence, by using the temperature field of the model as input, the stress distribution can be calculated, taking thermal stress into consideration. According to the theory of elasticity, when the temperature changes, the strain of an elastic body is composed of two parts: the thermal expansion strain and the strain caused by the mutual constraint of other parts. The stress-strain relation of the elastic body is modified as follows:

(12) $$\left\{ \begin{array}{c} \varepsilon_{x}=\frac{1}{E}\left[\sigma_{x}-v\left(\sigma_{y}+\sigma_{z}\right)\right]+\alpha T\\ \varepsilon_{y}=\frac{1}{E}\left[\sigma_{y}-v\left(\sigma_{x}+\sigma_{z}\right)\right]+\alpha T\\ \varepsilon_{z}=\frac{1}{E}\left[\sigma_{z}-v\left(\sigma_{x}+\sigma_{y}\right)\right]+\alpha T\\ \gamma_{\text{yz}}=\frac{2\left(1+v\right)}{E}\tau_{\text{yz}}\\ \gamma_{\text{xz}}=\frac{2\left(1+v\right)}{E}\tau_{\text{xz}}\\ \gamma_{\text{xy}}=\frac{2\left(1+v\right)}{E}\tau_{\text{xy}} \end{array}\right.$$

where σ refers to the normal stress, ε refers to the normal strain, v is Poisson's ratio, τ is the shearing stress and γ is the shearing strain. By using classic balanced differential equations, geometric equations, and the aforementioned physical equation, the stress field of the proposed model can be obtained.

**Fig. 4:**
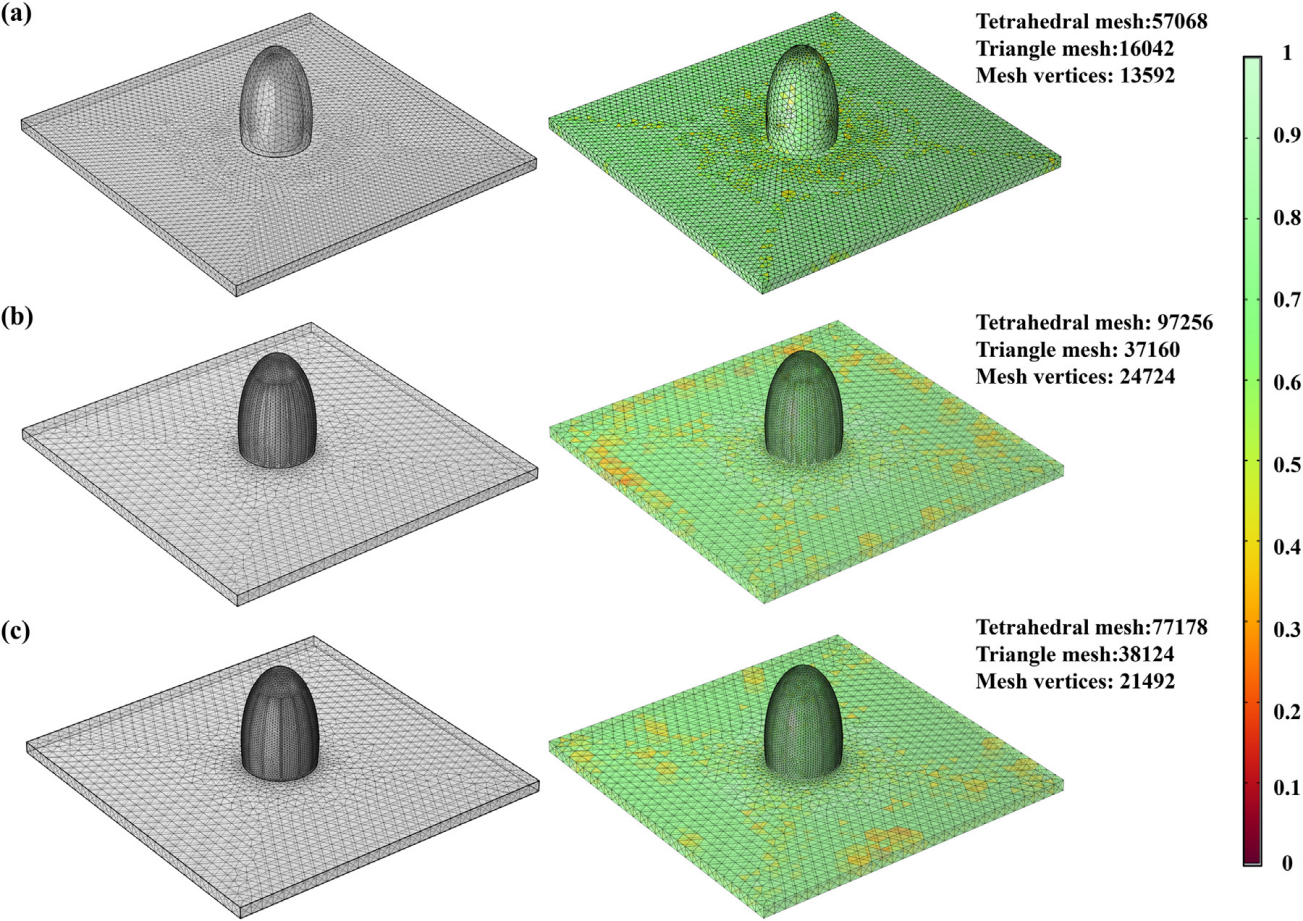
**Finite element mesh and grid quality analysis of three types of structures: (a) single layer structure, (b) line-shaped structure, (c) Z-shaped structure**.

This study utilized COMSOL for grid partitioning based on physical fields and for solving the proposed simulation model. The grid partitioning is illustrated in Fig. 4. The quality of the grid is displayed using color-coded meshes with the skewness indicator, mostly light green, indicating good quality grids. The numbers of tetrahedral meshes, triangle meshes, and mesh vertices are also labeled in the figure, demonstrating the level of mesh refinement.

## 3 Results and discussion

By following the aforementioned methodology, the temperature field and stress field of the proposed model can be obtained. To further evaluate the thermal performance of the shell structures, the temperature, heat loss, and thermal stress of these structures are compared and analyzed.

### 3.1 Thermal resistance and temperature field of the proposed structure

Before discussing the temperature field of the shell structures, the thermal resistance of the three proposed architectures needs to be quantified. The thermal resistance is defined as the ratio between the temperature difference at both ends of an object and the power of the heat source when heat is transmitted through the object.

(13) $$R=\frac{\Delta T}{P}$$

where R refers to the thermal resistance, ΔT is the temperature difference and P is the heat flow power.

**Fig. 5:**
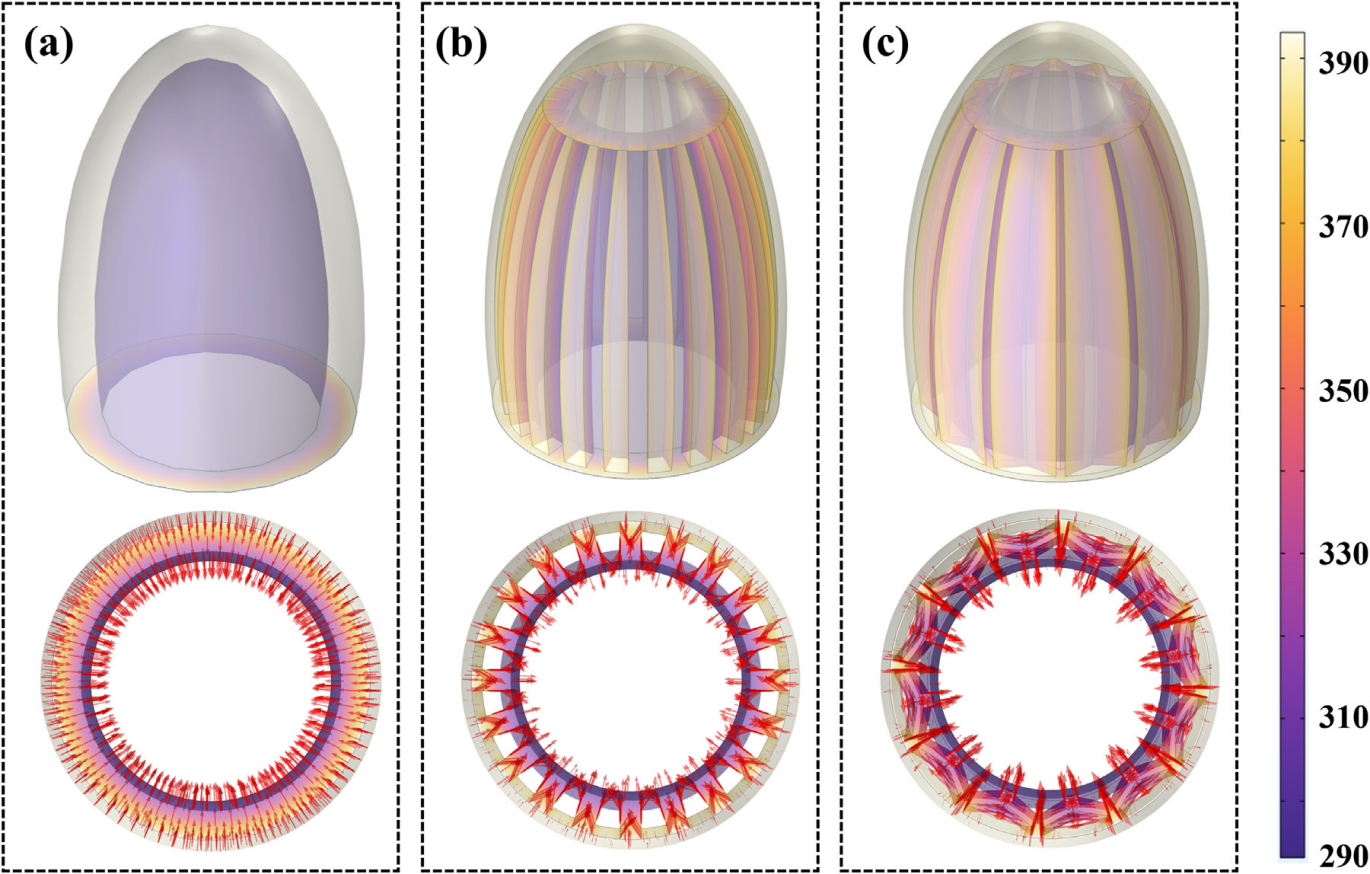
**Temperature field and heat conduction vector under a temperature difference of 100 K between the inside and outside: (a) single layer structure, (b) line-shaped structure, (c) Z-shaped structure**.

To investigate thermal resistance, three models were constructed, each containing only the shell structure itself without the regolith floor. Only thermal conduction was considered. The outer surface temperature of the structures was set to 393K, while the inner surface temperature was set to 293K, resulting in a constant temperature difference of 100K. The resulting temperature distribution and internal heat conduction vector are depicted in Fig. 5. Additionally, the heat exchange power was calculated and is presented in Table 4. The thermal resistances of the single layer structure, line-shaped structure, and Z-shaped structure were 0.0436W/K, 0.102W/K, and 0.227W/K, respectively. Therefore, it can be concluded that the Z-shaped structure exhibits superior thermal resistance compared to the other two shell structures.

**Table 4:** **Heat exchange power under a temperature difference of 100 K**.

	Single layer	Line-shaped structure	Z-shaped structure
Heat conduction	2294.30 W	978.11 W	440.96 W
Heat conduction and heat radiation	\	1691.90 W	−556.96 W

To explore the effect of radiation, the aforementioned model was modified to include interlayer radiation. This modification enabled the evaluation of the heat exchange rate, as shown in Table 4. Notably, the heat exchange power of the Z-shaped structure was negative, indicating the need for internal heating to maintain a steady state. This occurrence can likely be attributed to radiation leakage due to the absence of a floor in the shell structure. Regardless, it can be acknowledged that the effect of radiation cannot be overlooked.

**Fig. 6:**
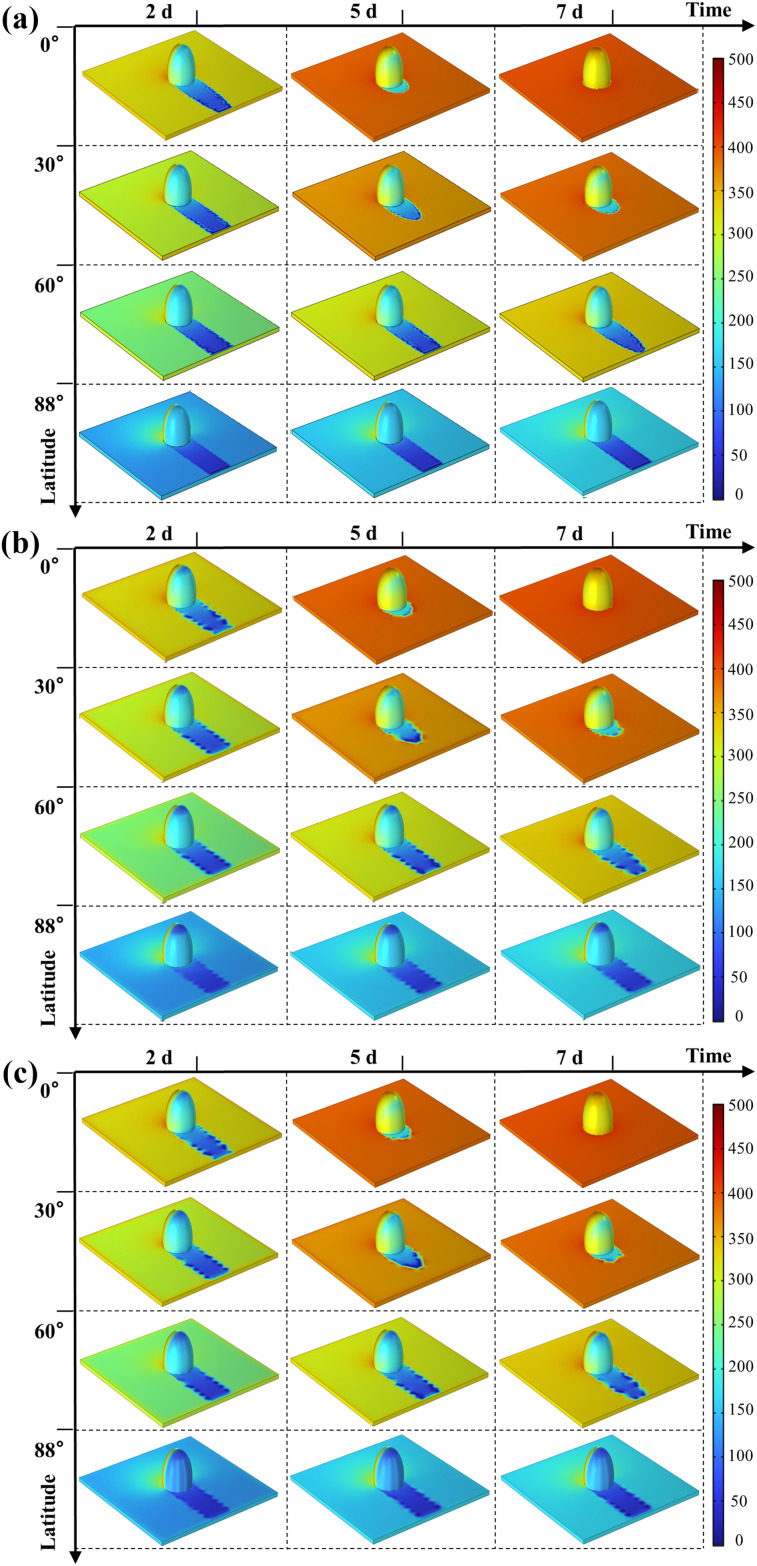
**Temperature field of the proposed structure: (a) single layer structure, (b) line-shaped structure, (c) Z-shaped structure**.

Fig. 6 displays the temperature distribution on the shell structures at various locations and times. In this context, "7 d" signifies noon at each latitude. Upon observing the cuboid surface of the regolith, several noteworthy analyses can be performed. First, there are areas of reduced temperature caused by the shielding of external radiation by the upright shell structure. The location and size of these shadow areas change with both time and location. Since shell structures receive thermal radiation from all sides of the regolith surface and thermal conduction from adjacent regolith, low-temperature shadow areas can also influence shell structures through thermal conduction and radiation. Consequently, it can be affirmed that the model considers self-shadowing effects, thereby enhancing the accuracy of the simulation results. Second, the shadowed areas are not consistent and feature flat edges, particularly in the case of double-shell structures. It can be inferred that the uneven temperature distribution on the shell surface leads to uneven thermal radiation toward the lunar surface, resulting in the formation of nonuniform shadow areas.

From the observation of the shell structures, the following analyses are related. First, there is a high-temperature zone facing the radiation direction and a low-temperature zone facing the back radiation direction in the structure. The temperature difference on the two sides of the structure can be the main reason for the large thermal stress, which can cause damage to the structure. Second, for a single-layer structure, the temperature distribution of the low-temperature zone is relatively uniform compared to that of double-shell structures, which can be contributed by the uniform architecture of the single-layer structure. Clearly, highlighted contour lines can be seen in the low-temperature zone of the Z-shaped structure at high latitudes. These contour lines are located at the connection between the middle layer and the outer layer, the junction between the roof and the main structure, and the intersection line of radiation incidence with the shell structure. This is expected because the junction part causes a sharp change in the amount of thermal conduction and the intersection line causes a sharp change in the external irradiance. Another interesting phenomenon is the low temperature of the catenary roof in double-shell structures. This is due to the radiation isolation of the roof's inner surface.

For a more quantitative expression, the temperature extremes of the structure are listed in Table S2. It shows that a larger thermal resistance of a structure results in a higher highest temperature and lower lowest temperature, leading to a high ΔT. This trend is reasonable because a structure with a large thermal resistance can hinder the energy input from high-temperature outside areas, as well as the energy output to low-temperature outside areas. Additionally, the temperature of the structure surface varies greatly from the normal regolith temperature. This indicates the importance of considering the radiation from the lunar regolith surface, as well as the self-shadowing effect.

### 3.2 Heat loss analysis

In the proposed simulation model, the innermost surface of the shell structure is maintained at a constant temperature of 293.5 K through internal cooling or heating regulation. Analyzing the heat loss through the innermost surface provides valuable insights into building energy consumption. A positive heat loss indicates that the internal “air condition” is functioning to cool the structure, while a negative value suggests heating.

**Fig. 7:**
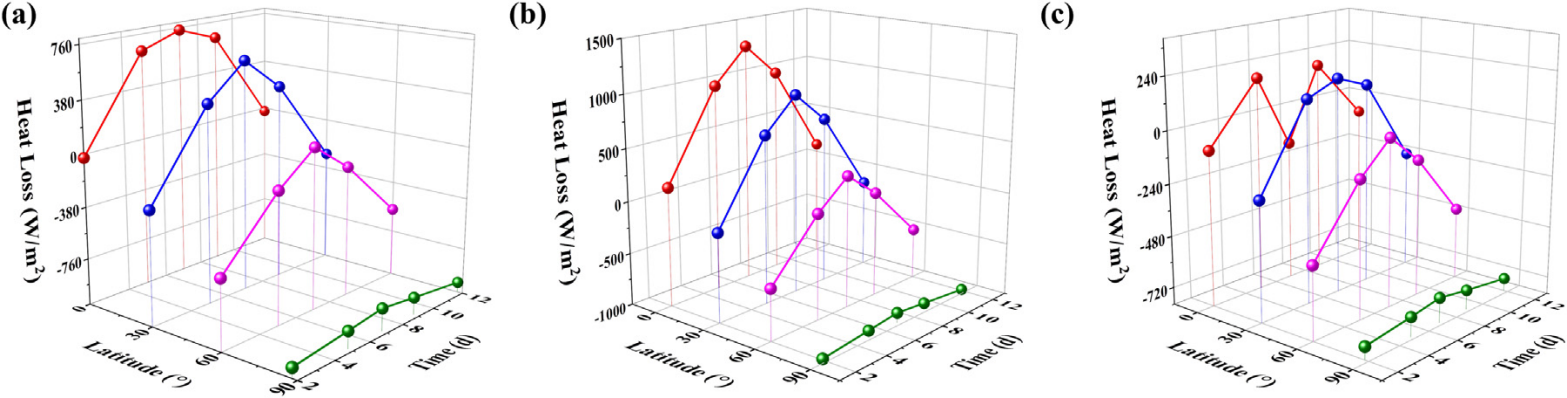
**Heat loss of 3 types of shell structures: (a) single layer structure, (b) line-shaped structure, (c) Z-shaped structure**.

For a comprehensive comparison, the heat loss results for the three structures are presented in Fig. 7. To enhance clarity, the heat loss at 9 d and 12 d is also included, which is equivalent to that at 5 d and 2 d, respectively.

Several clear trends can be observed in the heat loss indicator. First, as latitude increases, heat loss decreases (it should be specified that negative values are smaller than positive values). This implies that structures at higher latitudes require more internal heating. Second, as time progresses from the morning to noon, heat loss tends to decrease, reflecting the natural increase in temperature and the corresponding need for cooling. Last, among the three structures, the range of heat loss for the Z-shaped structure is apparently smaller, namely, −741.18W to 197.16W, which is due to the high thermal resistance performance.

**Fig. 8:**
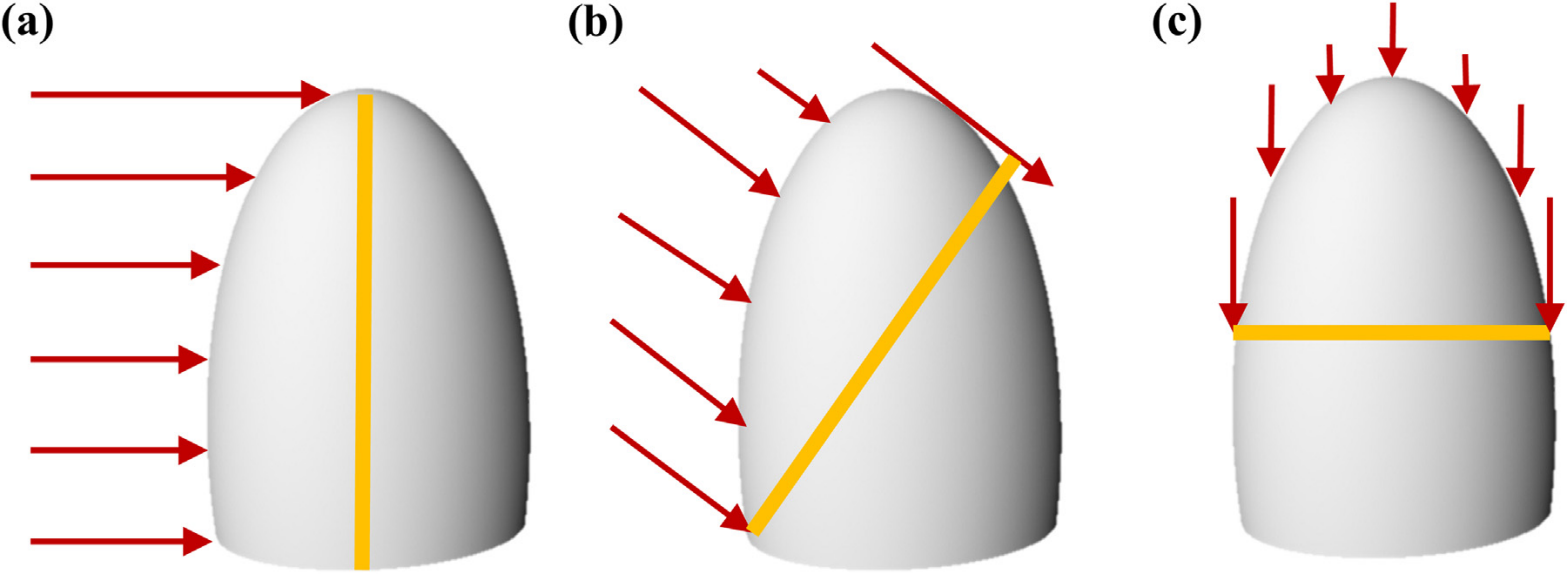
**Radiation incidence angle and effective incidence area: (a) parallel incidence, (c) oblique incidence, (c)vertical incidence**.

A counterintuitive phenomenon can be observed in the summary figure. The heat loss of the Z-shaped structure at the equator and at noon is negative, indicating that the inside “air condition” provides heating. This is a result of the combined effects of the structure's large thermal resistance, the reduced effective radiation incident area, and the radiation isolation on the inner surface of the catenary. First, the Z-shaped structure exhibits the highest thermal resistance, resulting in insufficient heat transfer to the structure's interior, thus facilitating this unique phenomenon. Second, the effective radiation incident area is smallest for the egg-shaped structure, as illustrated in Fig. 8. The red arrows represent incident radiation, and the yellow line represents the effective radiation incident length, which is the shortest under vertical incidence. Consequently, while receiving vertical incidence, the amount of radiation is the smallest. Finally, most of the external radiation is absorbed by the catenary roof. However, the inner surface of the catenary roof is set to unable to transfer heat through radiation. This not only hampers the efficiency of heat transfer but also disrupts the balance of cavity radiation inside the structure, leading ultimately to the counterintuitive phenomenon.

**Fig. 9:**
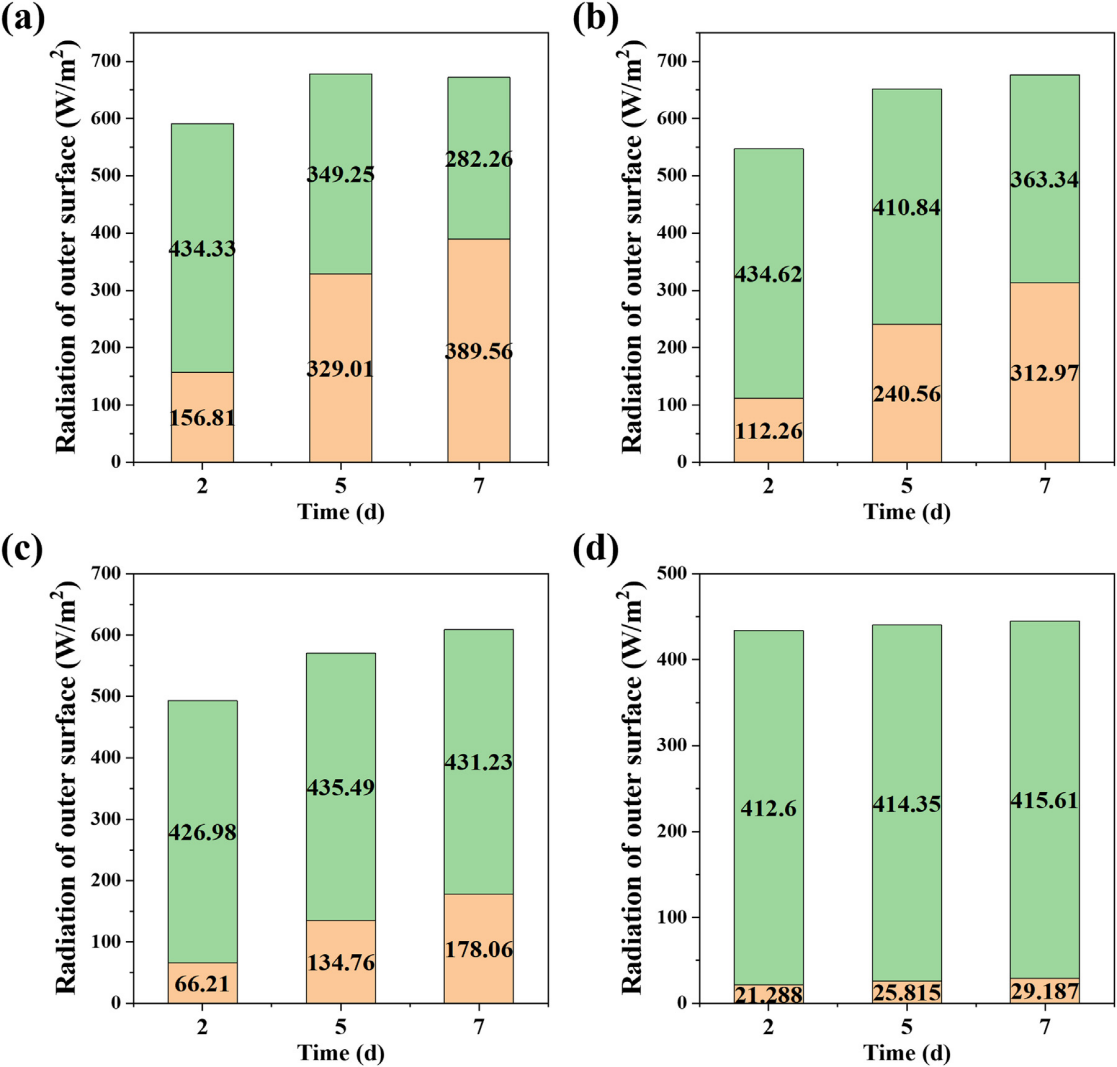
**Radiation received by the outer surface of the Z-shaped structure: (a) equator, (b) 30° latitude, (c) 60° latitude, (d) 88° latitude.** The green part refers to external radiation, and the yellow part refers to mutual radiation.

For a more quantitative comparative analysis, the radiation amount received by the outer surface of the Z-shaped structure is depicted and labeled in Fig. 9. As discussed in Eq. (8), the total radiation consists of external radiation, mutual radiation, and ambient radiation. In the proposed simulation model, the ambient radiation is assumed to be zero. The mutual radiation received by the outer surface originates from the radiation of the surrounding regolith, which increases with the temperature of the regolith. Consequently, its value increases as the position nears the equator and the time approaches noon. The external radiation source, specifically solar irradiance and Earth irradiance, exhibits a constant heat flux, with the effective incidence area being the determining factor. Fig. 9 does not show any clear trends in the external radiation due to the irregular shape of the egg-shaped structure and the absence of a monotonic relationship between the incident angle and effective area. However, the overall radiation amount at the equator decreases at noon, thus validating the aforementioned explanation. Furthermore, the figure clearly indicates that the external radiation in the polar region surpasses the mutual radiation, which serves as the primary influencing factor on the temperature of the outer surface of the shell structure.

Consequently, it is recommended that habitat structures and energy storage structures at high latitudes possess a larger longitudinal cross-sectional area to maximize solar irradiance absorption. The specific shape of the structure can be further tailored to the specific latitude. Additionally, the use of construction materials with selective absorption properties on the sidewalls can maximize heat radiation absorption and minimize heat release.

### 3.3 Thermal stress analysis

**Fig. 10:**
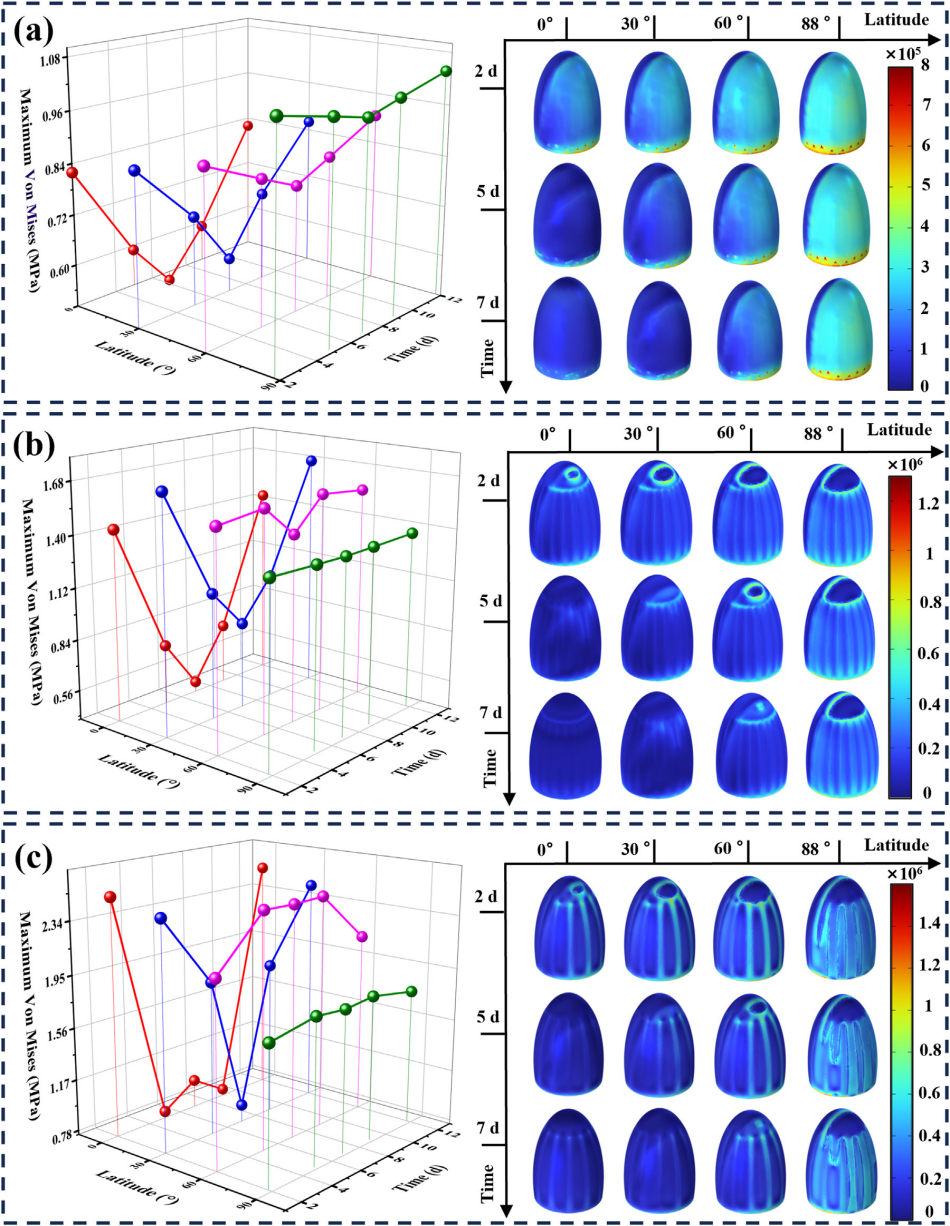
**Thermal stress of 3 types of shell structures: (a) single layer structure, (b) line-shaped structure, (c) Z-shaped structure**.

Thermal stress arises due to the constraint that impedes the unrestricted thermal expansion of construction materials. In the face of lunar temperature fluctuations, thermal stress can be substantial and variable, leading to fatigue and damage in building materials. The thermal stress field of the shell structures is calculated and depicted in Fig. 10 following the coupled thermomechanical analysis. The left section shows a three-dimensional line chart illustrating the maximum von Mises stress under different conditions. On the right side, a heatmap represents the von Mises stress in the shell structures. It is important to note that the legend of the heatmaps has been adjusted to enhance the visibility of the stress distribution. Specifically, considering the single layer structure as an example, the maximum von Mises stress is 1.04MPa. However, the range of the heatmap is reduced to [0,0.8MPa]. The units whose stress is greater than 0.8MPa are all marked with the deepest red color.

In most instances, the maximum stress is concentrated in the connection between the shell structure and the lunar regolith cuboid. The single structure exhibits a maximum von Mises stress of 1.04MPa at latitude $88^{\circ }$ on 2d. The maximum von Mises stress of the line-shaped structure is 1.68MPa, which occurs at latitude $30^{\circ }$ on 2d. In contrast, the Z-shaped structure experiences a maximum von Mises stress of 2.52MPa at the equator on 2d. The thermal stress in the Z-shaped structure consistently surpasses that in the line-shaped structure, not to mention the single-layer structure. This can be attributed to two factors. First, the Z-shaped structure undergoes greater temperature variations due to its excellent thermal insulation performance. Second, the Z-shaped structure is more susceptible to stress concentration because its interlayers have fewer fulcrums and fewer areas of connection. Nevertheless, the largest stress value remains below the strength threshold of most regolith-based extraterrestrial construction materials, indicating that the Z-shaped structure is safe.

The field of thermal stress is closely associated with the temperature field. In the case of a single layer structure, there is a uniform stress distribution that correlates with the temperature field. Additionally, the maximum thermal stress increases with latitude and time. However, this trend is not consistent for line-shaped and Z-shaped structures, which can be attributed to their complex architectural design. Regarding double-shell structures, Section 3.1 discusses that the areas with the highest temperature in the temperature field are located at the connections between layers, the junction between the roof and the main structure, and the intersection line of radiation incidence. This same pattern is observed in the thermal stress field due to the significant temperature differences in these areas. Furthermore, the connection and junction areas are high-stress concentration regions because they involve sharp changes in structural shape.

Based on the aforementioned discussion, the following suggestions can be made to mitigate large and variable thermal stresses. First, structures with high thermal resistance tend to exhibit large temperature differences, resulting in significant thermal stress. Therefore, the use of elastic construction materials with low and similar coefficients of thermal expansion is recommended for such structures. Second, when designing lunar base structures that are exposed to extreme temperature variations [45], it is crucial to avoid sharp shape change regions, such as unnecessary gaps, holes, and grooves. Additionally, connections with different curvatures should be designed as smoothly as possible to minimize stress concentration.

## 4 Conclusion

In conclusion, this study employed classical thermomechanical coupling simulations to develop a comprehensive methodology for evaluating the performance of lunar shell structures. This methodology incorporated generalized characterization patterns for extraterrestrial construction materials, assumed settings for key loads and boundary conditions, and considered the effect of structure self-shadowing. By applying this method, 36 working situations of shell structures were simulated, including three types of structures, four latitudes, and three time nodes. The temperature field, heat loss, and thermal stress were calculated and analyzed for each situation. The main conclusions drawn from this study are as follows:

(1) The design of a hollow multilayer can effectively enhance the thermal insulation performance of a shell structure. Compared to a single-shell structure, the design of line-shaped interlayers increases the thermal resistance by 2.34 times, while the design of Z-shaped interlayers increases it by 5.2 times. Consequently, even under lunar temperature shock, the heat loss of Z-shaped structures is reduced to a range of −741.18W to 197.16W.
(2) Shell structures located at high latitudes receive significantly higher levels of radiance ($415.61\;W/m^{2}$) from solar irradiance than from neighboring regolith ($29.19\;W/m^{2}$). For this reason, it is advisable to design these structures with larger longitudinal cross-sectional areas and construct them using selective absorption materials. Additionally, the structure shape can be further tailored to a specific latitude to increase the effective radiation area.
(3) Under extreme temperature variations, the phenomenon of stress concentration can become severe. In the case of Z-shaped structures, the maximum von Mises stress is 2.5 times greater than that of single structures, with higher stress concentrations occurring at the connection, junction, and intersection areas. Sharp shape changes in the architectural design of the structure should be avoided. Moreover, the connections of curves should be as smooth as possible.

## Declaration of competing interest

The authors declare that they have no conflicts of interest in this work.

## Appendix Supplementary materials

Image, application 1
