# Supplementary material for: Performance of lunar shell structure for moonbase subjected to low gravity coupled with changing temperature
Source: Fundam Res. 2024 Sep 3;6(3):1691–703. doi: 10.1016/j.fmre.2024.08.005 (PMC13247460; doi:10.1016/j.fmre.2024.08.005)
Supplement: Supplementary file 1 [file mmc1.docx]

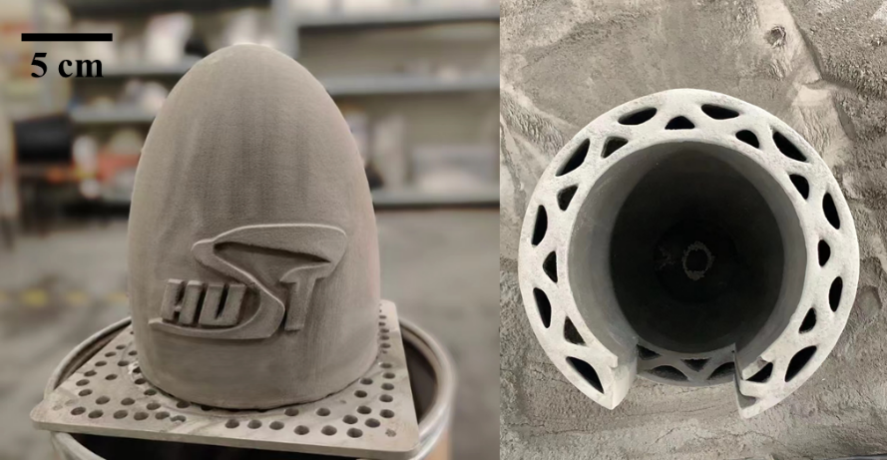


Fig. S1 Scaled shell structure manufactured by selective laser sintering.

Table S1 Summary of thermal loads

| Region | Time | Irradiance angle (°) | | Temperature of lunar surface (K) | Lunar heat flow (W/m^2^) |
| --- | --- | --- | --- | --- | --- |
|  |  | Solar radiation, 1368W | Earth radiation, 0.15W |  |  |
| Equator | 14-28d (Night) | 90 | 0 | 120 | 0.015 |
|  | 2d/12d | 64.3 | 0 | 342.96 | 0.015 |
|  | 5d/9d | 25.7 | 0 | 387.31 | 0.015 |
|  | 7d (Noon) | 0 | 0 | 394.1 | 0.015 |
| 30° Latitude | 14-28d (Night) | 90 | 30 | 120 | 0.015 |
|  | 2d/12d | 72.9 | 30 | 330.88 | 0.015 |
|  | 5d/9d | 47.1 | 30 | 373.66 | 0.015 |
|  | 7d (Noon) | 30 | 30 | 380.2 | 0.015 |
| 60° Latitude | 14-28d (Night) | 90 | 60 | 120 | 0.015 |
|  | 2d/12d | 81.4 | 60 | 288.50 | 0.015 |
|  | 5d/9d | 68.6 | 60 | 325.71 | 0.015 |
|  | 7d (Noon) | 60 | 60 | 331.4 | 0.015 |
| South Pole | 14-28d (Night) | 90 | 88 | 120 | 0.015 |
|  | 2d/12d | 89.4 | 88 | 152.26 | 0.015 |
|  | 5d/9d | 88.6 | 88 | 167.81 | 0.015 |
|  | 7d (Noon) | 88 | 88 | 170.3 | 0.015 |

Table S2 Temperature analysis of the proposed structure

|  |  |  | $T_{max}$ | $T_{min}$ | $\Delta T$ | $T_{regolith}$ |
| --- | --- | --- | --- | --- | --- | --- |
| Single-shell structure | Latitude 0° | 2 d/12 d | 418.09 | 150.55 | 267.54 | 342.96 |
|  |  | 5 d/9 d | 407.73 | 183.96 | 223.77 | 387.31 |
|  |  | 7 d | 397.28 | 223.05 | 174.23 | 394.10 |
|  | Latitude 30° | 2 d/12 d | 418.45 | 146.67 | 271.78 | 330.88 |
|  |  | 5 d/9 d | 414.12 | 167.15 | 246.97 | 373.66 |
|  |  | 7 d | 410.17 | 180.52 | 229.65 | 380.20 |
|  | Latitude 60° | 2 d/12 d | 416.59 | 143.86 | 272.73 | 288.50 |
|  |  | 5 d/9 d | 418.57 | 148.37 | 270.2 | 325.71 |
|  |  | 7 d | 417.02 | 153.75 | 263.27 | 331.40 |
|  | Latitude 88° | 2 d/12 d | 412.54 | 141.95 | 270.59 | 152.26 |
|  |  | 5 d/9 d | 413.05 | 142.13 | 270.92 | 167.81 |
|  |  | 7 d | 413.42 | 142.27 | 271.15 | 170.30 |
| Double-shell structure with line-shaped interlayer | Latitude 0° | 2 d/12 d | 424.04 | 110.29 | 313.75 | 342.96 |
|  |  | 5 d/9 d | 410.54 | 187.11 | 223.43 | 387.31 |
|  |  | 7 d | 393.93 | 232.08 | 161.85 | 394.10 |
|  | Latitude 30° | 2 d/12 d | 423.93 | 98.71 | 325.22 | 330.88 |
|  |  | 5 d/9 d | 417.3 | 146.24 | 271.06 | 373.66 |
|  |  | 7 d | 412.88 | 183.14 | 229.74 | 380.20 |
|  | Latitude 60° | 2 d/12 d | 421.36 | 87.92 | 333.44 | 288.50 |
|  |  | 5 d/9 d | 424.30 | 104.04 | 320.26 | 325.71 |
|  |  | 7 d | 423.19 | 116.91 | 306.28 | 331.40 |
|  | Latitude 88° | 2 d/12 d | 416.78 | 73.26 | 343.52 | 152.26 |
|  |  | 5 d/9 d | 417.31 | 75.41 | 341.9 | 167.81 |
|  |  | 7 d | 417.69 | 76.74 | 340.95 | 170.30 |
| Double-shell structure with Z-shaped interlayer | Latitude 0° | 2 d/12 d | 426.21 | 110.05 | 316.16 | 342.96 |
|  |  | 5 d/9 d | 411.26 | 170.06 | 241.2 | 387.31 |
|  |  | 7 d | 393.94 | 223.97 | 169.97 | 394.10 |
|  | Latitude 30° | 2 d/12 d | 426.40 | 98.19 | 328.21 | 330.88 |
|  |  | 5 d/9 d | 420.04 | 142.97 | 277.07 | 373.66 |
|  |  | 7 d | 414.13 | 162.83 | 251.3 | 380.20 |
|  | Latitude 60° | 2 d/12 d | 424.11 | 87.11 | 337 | 288.50 |
|  |  | 5 d/9 d | 426.62 | 103.70 | 322.92 | 325.71 |
|  |  | 7 d | 425.19 | 116.70 | 308.49 | 331.40 |
|  | Latitude 88° | 2 d/12 d | 419.60 | 70.49 | 349.11 | 152.26 |
|  |  | 5 d/9 d | 420.16 | 73.09 | 347.07 | 167.81 |
|  |  | 7 d | 420.56 | 74.74 | 345.82 | 170.30 |

$T_{max}$ refers to the highest temperature on the outer surface of the shell structure, and $T_{min}$ refers to the lowest temperature. The location of $T_{max}$ lies on the surface facing the external irradiance, and the location of $T_{min}$ lies on the surface backing it. The difference between $T_{max}$ and $T_{min}$ is listed below. The temperature of the normal lunar regolith, derived from Eq. 2-7, is also included as a reference.
